# *Wolbachia*-Mediated Resistance to Dengue Virus Infection and Death at the Cellular Level

**DOI:** 10.1371/journal.pone.0013398

**Published:** 2010-10-15

**Authors:** Francesca D. Frentiu, Jodie Robinson, Paul R. Young, Elizabeth A. McGraw, Scott L. O'Neill

**Affiliations:** 1 School of Biological Sciences, The University of Queensland, Brisbane, Australia; 2 Centre for Infectious Disease Research, School of Chemistry and Molecular Biosciences, The University of Queensland, Brisbane, Australia; Institut Pasteur, France

## Abstract

**Background:**

Dengue is currently the most important arthropod-borne viral disease of humans. Recent work has shown dengue virus displays limited replication in its primary vector, the mosquito *Aedes aegypti*, when the insect harbors the endosymbiotic bacterium *Wolbachia pipientis*. *Wolbachia*-mediated inhibition of virus replication may lead to novel methods of arboviral control, yet the functional and cellular mechanisms that underpin it are unknown.

**Methodology/Principal Findings:**

Using paired *Wolbachia*-infected and uninfected *Aedes*-derived cell lines and dengue virus, we confirm the phenomenon of viral inhibition at the cellular level. Although *Wolbachia* imposes a fitness cost to cells via reduced proliferation, it also provides a significant degree of protection from virus-induced mortality. The extent of viral inhibition is related to the density of *Wolbachia* per cell, with highly infected cell lines showing almost complete protection from dengue infection and dramatically reduced virus titers compared to lines not infected with the bacteria.

**Conclusions/Significance:**

We have shown that cells infected with *Wolbachia* display inhibition of dengue virus replication, that the extent of inhibition is related to bacterial density and that *Wolbachia* infection, although costly, will provide a fitness benefit in some circumstances. Our results parallel findings in mosquitoes and flies, indicating that cell line models will provide useful and experimentally tractable models to study the mechanisms underlying *Wolbachia*-mediated protection from viruses.

## Introduction

Dengue is the most important arthropod-borne viral disease currently affecting human populations. The severity and frequency of dengue outbreaks is rapidly increasing across the world and the geographic range of the virus is expanding from the tropics into more temperate areas [1], [2], [3]. Dengue fever and its often lethal complication, dengue hemorrhagic fever, have become the leading arboviral causes of illness and death in recent years [3], [4]. Dengue virus (DENV) is transmitted to humans by *Aedes* mosquitoes, with the most important vectors being *Aedes aegypti* and, to a lesser extent, *Aedes albopictus*. The absence of either a vaccine or therapeutic strategies against all four DENV (DENV 1–4) serotypes has reinforced the need for improved approaches to control vector populations.

Infection of mosquitoes with the maternally inherited bacterial endosymbiont *Wolbachia pipientis* has been proposed as a novel strategy to modify mosquito populations and their subsequent ability to transmit pathogens [5]. *Wolbachia* infection of mosquitoes may reduce transmission of DENV in several ways. *Aedes aegypti* females infected with the *w*MelPop-CLA strain of *Wolbachia* have dramatically reduced life spans [6]. Eliminating older mosquitoes in a population may break the transmission cycle of DENV since the virus requires 7–14 days to complete its extrinsic incubation period in the vector, a period representing a substantial proportion of the mosquito lifespan [7]. Importantly, *Wolbachia* infected mosquitoes also show reduced vector competence [8] and reduced replication of dengue virus [8], [9], Chikungunya virus [8], [9], *Plasmodium* [8] and filarial nematodes [10]. More generally, *Drosophila melanogaster* flies infected with *Wolbachia* show significantly reduced replication of RNA viruses [11], [12], with this effect strongest in *Wolbachia* strains most closely related to the strain *w*MelPop [13].

*Wolbachia*-induced resistance to RNA virus infection in insects may provide us with a powerful way to control insect-transmitted diseases yet the cellular and molecular mechanisms that underpin resistance remain unknown. *Wolbachia* may induce priming of insect defense genes prior to virus infection [8], [9], [10], [14]. A range of immune genes was found to be upregulated in *w*MelPop-CLA infected versus uninfected mosquitoes [8], [10], as well as in *Aedes aegypti* infected with a different *Wolbachia* strain, *w*AlbB [9]. Alternatively, *Wolbachia* may directly interfere with virus replication at the cellular level and/or may directly or indirectly compete for host resources [8], [14] since both bacteria and virus require the same environment (the cytoplasm) in which to replicate [15], [16], [17]. Spatial exclusion of DENV from *Wolbachia*-infected tissue and cells has been shown via immunofluorescence microscopy of mosquito sections [8].

Understanding the mechanism of viral inhibition using whole organisms is complicated by *Wolbachia* density differences among various tissues [8], [15] and the dynamic nature of these infections [16], [18]. In addition, the insect host genetic background, age and environment also have a significant effect on *Wolbachia* infection levels [19], [20], [21]. By contrast, cell line models circumvent many of these complications, as well as being more experimentally tractable. Examining the interaction between *Wolbachia* and DENV in mosquito cell lines will determine whether they provide good models for dissecting the mechanistic basis of viral inhibition.

Here, we explored the replication dynamics of the New Guinea C strain of DENV serotype 2 (DENV-2) in two sets of paired *Wolbachia*-infected and uninfected mosquito cell lines, RML12 and C6/36, both originally from *Aedes albopictus*. The C6/36 line was stably infected with the *w*MelPop-CLA strain, that had been previously adapted to RML12 cells over the course of more than 250 passages. Both cell lines displayed almost 100% infection with *Wolbachia* but they differed dramatically in the density of bacteria per cell. Although *Wolbachia* infection had a negative effect on cell proliferation, suggesting a heavy metabolic cost it also conferred a significant degree of protection against DENV-2 induced cytopathic effects. DENV-2 replication was dramatically reduced in both *Wolbachia*-infected lines compared to uninfected controls, with an almost complete absence of viral replication in the cell line most densely infected with *Wolbachia*.

## Results

### *Wolbachia* infection and density in two insect cell lines

**Figure 1:**
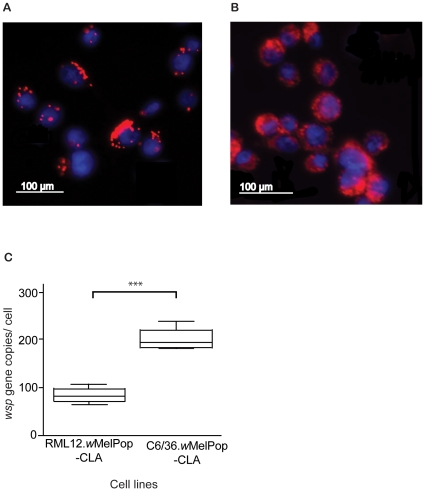
*Wolbachia* infections in the RML12.wMelPop-CLA (A) and C6/36.wMelPop-CLA (B) cell lines, visualized using fluorescent in situ hybridization (FISH) with *Wolbachia*-specific probes. *Wolbachia* bacteria are shown in red and cell nuclei are shown in blue (stained with DAPI). (C) Differences in the absolute density of *Wolbachia* per cell between the RML12.wMelPop-CLA and C6/36.wMelPop-CLA lines. Box and whisker plots display medians and 5 and 95 percentiles of five replicate pools of 2.5×105 cells/line (*** p<0.001 by t-test).

We used the *Aedes albopictus* [22] RML12 cell line previously infected with *w*MelPop-CLA [23] (here termed RML12.*w*MelPop-CLA; Figure 1A). A control *Wolbachia* uninfected RML12 line, named RML12.Tet, was obtained after three rounds of tetracycline treatment, as checked by fluorescent *in situ* hybridization (FISH) and PCR. The *Ae. albopictus* cell line C6/36, routinely used in DENV studies, was also infected with *w*MelPop-CLA purified from the RML12.*w*MelPop-CLA using a modified shell vial technique [24]. By passage 6, 100% (±0% s.e.) of cells counted in the C6/36.*w*MelPop-CLA line, visualized using FISH, were infected with *Wolbachia*. Stable infection with *w*MelPop-CLA remained at 100% throughout the course of the experiments, as verified by FISH (Figure 1B). *Wolbachia* infection rates in the RML12.*w*MelPop-CLA line were determined to be 94.50% (±1.59% s.e.), based on counts of cells visualized using FISH.

Next, we examined the density of *Wolbachia* per cell in the two infected cell lines by first examining the ratio of number of copies of the *wsp* (*Wolbachia* surface protein) gene relative to the mosquito reference gene *RpS17* [7] using quantitative PCR (qPCR). Because some cell lines have altered levels of ploidy [25] and it cannot be assumed that reference genes are represented by single copies, we also quantified the absolute number of copies of the *wsp* gene by qPCR [8]. A significant difference in *Wolbachia* density between cell lines was found by examining the expression of the *wsp* gene relative to the expression of the mosquito housekeeping gene *RpS17* [7] (Wilcoxon rank sum test W = 25, p = 0.008). Quantification of the absolute, rather than relative, number of copies of the *wsp* gene also showed that the C6/36.*w*MelPop-CLA line had, on average, significantly more bacteria per cell than the RML12.*w*MelPop-CLA line (t = 11.5751, df = 8, p<0.001). The average density of *Wolbachia* per cell in the C6/36.*w*MelPop-CLA line was approximately three times higher than the density in the RML12.*w*MelPop-CLA line (Figure 1C).

### *Wolbachia*-infected cells proliferate slowly but resist DENV-induced mortality

**Figure 2:**
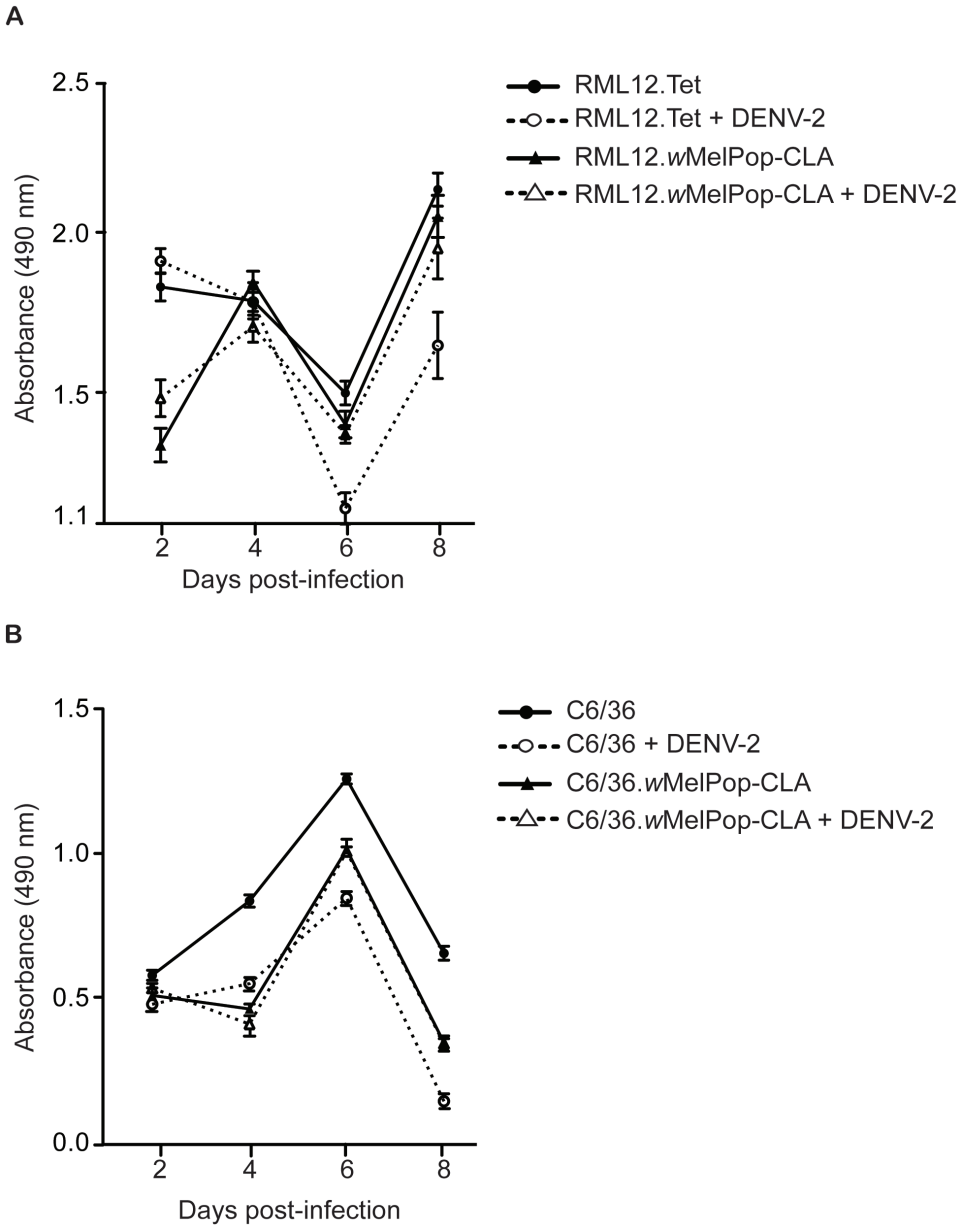
Cell proliferation of the RML12.wMelPop-CLA (A) and C6/36.wMelPop-CLA (B) lines at days 2, 4, 6 and 8 after infection with DENV-2 (NGC strain) or mock infection (no DENV-2). Bars indicate means ± SEM of absorbance measurements from 8 replicate wells.

The *w*MelPop-CLA strain dramatically reduces mosquito fitness through reduction in lifespan [6] and impaired probing and feeding [26], [27]. We investigated whether cells infected with *w*MelPop-CLA also displayed reduced fitness by assaying cell proliferation at 4 time points over the course of 8 days. Both *Wolbachia*-infected cell lines showed reduced cell proliferation compared to uninfected cell lines (Figure 2), although to differing extents. In the RML12.*w*MelPop-CLA cell line, cell proliferation was particularly low by day 2 (Figure 2A). However, by day 4 post-infection, RML12.*w*MelPop-CLA cells had recovered and closely followed the proliferation pattern of RML12.Tet cells. Cells from the C6/36.*w*MelPop-CLA line displayed a dramatically lower rate of proliferation compared to C6/36 cells at all four time points, suggesting a sustained metabolic cost to *Wolbachia* infection (Figure 2B).

Next, we investigated the effect of *Wolbachia* infection on proliferation when cells were also infected with DENV-2. At days 6 and 8, RML12.Tet cells that are infected with DENV-2 proliferate far more slowly than RML12.*w*MelPop-CLA that are also infected with virus (Figure 2A). Two-way ANOVA results for each day indicated that dual infection with *Wolbachia* and DENV significantly impacted on cell proliferation only on days 6 and 8 (day 6: *SS* = 0.22, *F*_1, 28_ = 13.63, *P* = 0.001; day 8: *SS* = 0.48, *F*_1, 28_ = 10.86, *P* = 0.003). A more dramatic reduction in cell proliferation was found for the C6/36 cells infected with DENV-2 when compared to virus infected C6/36.*w*MelPop-CLA cells (Figure 2B). Interestingly, the degree of proliferation in the C6/36.*w*MelPop-CLA cells was the same regardless of whether they were infected with DENV-2 or not, suggesting virus infection had no effect on *Wolbachia*-infected cells. By contrast, DENV-2 infection had a significant negative effect on cell proliferation in the C6/36 line only, at days 4, 6 and 8 post-infection (day 4: SS = 0.01, *F*_1, 27_ = 28.55, P<0.001; day 6: SS = 0.18, *F*_1, 22_ = 45.94, P<0.001; day 8: SS = 0.42, *F*_1, 27_ = 124, P<0.001). In summary, although both C6/36.*w*MelPop and RML12.*w*MelPop lines experienced reduced cell proliferation compared to *Wolbachia*-uninfected counterparts, they were afforded a significant degree of protection from costs associated with DENV-2 infection.

### Reduced DENV-2 replication in *Wolbachia*-infected versus uninfected cells

**Figure 3:**
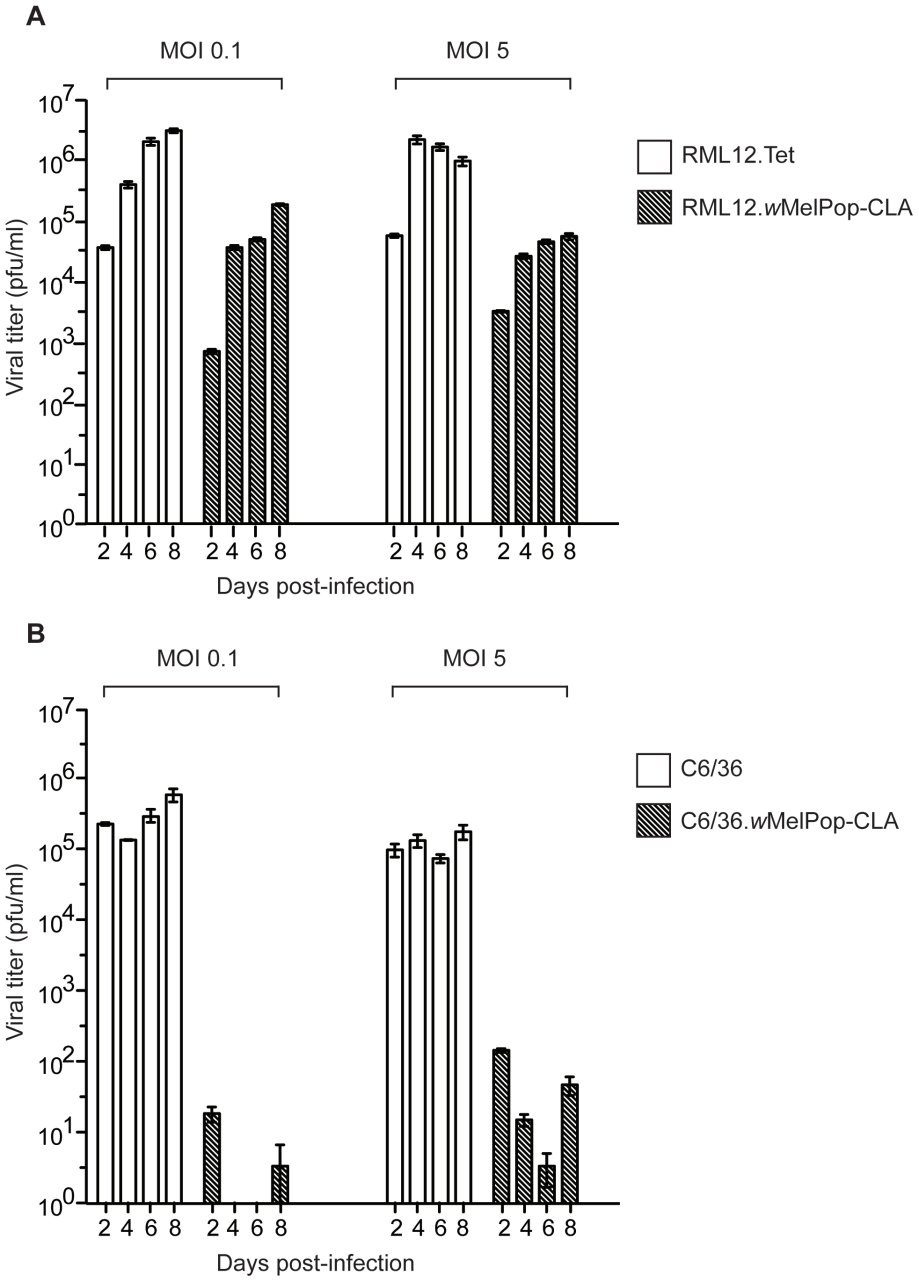
Viral replication of DENV-2 (NGC strain) in the RML12.wMelPop-CLA (A) and C6/36.wMelPop-CLA (B) cell lines harvested on days 2, 4, 6 and 8 post-infection, at multiplicities of infection of 0.1 and 5 (data for MOI 1 not shown). Bars indicate means ± SEM of viral titer from 3 replicate wells.

We next tested whether *Wolbachia*-infected cells showed reduced replication of DENV-2, as has been found in mosquitoes [8], [9]. Viral replication was dramatically reduced in both RML12.*w*MelPop-CLA and C6/36.*w*MelPop-CLA cell lines compared to lines not carrying *Wolbachia* (Figure 3), based on estimates of titer from virus harvested at days 2, 4, 6 and 8 post-infection. In RML12.*w*MelPop-CLA, at the three multiplicities of infection (MOIs) of 0.1, 1 and 5, and across all days sampled, viral titer was reduced by at least 1 log compared to RML12.Tet (Figure 3A; data for MOI 1 not shown). The results suggest that even at a high MOI, *w*MelPop-CLA had a strong inhibitory effect on virus growth. Although viral titer was markedly reduced, the inhibitory effect was not complete. By day 8 post-infection, at the low MOI of 0.1, at least 4.2 log pfu/ml was being produced in the RML.*w*MelPop-CLA cells (Figure 3A). ANOVA results indicated that *Wolbachia* infection, day post-infection and initial MOI all had highly significant but complex and interacting effects on viral titer (results not shown), with the largest effect due to the presence or absence of *Wolbachia*. Two-sample t-tests indicated that *Wolbachia* had a highly significant effect on viral output each day (day 2: t = 14.064, p<0.001; day 4: t = 10.799, p<0.001; day 6: t = 22.185, p<0.001; day 8: t = 11.214, p<0.001).

Viral titer in the C6/36.*w*MelPop-CLA line was even more dramatically reduced compared to the C6/36 control line, with the highest titer produced by *Wolbachia*-infected cells being 100 pfu/ml (Figure 3B; data for MOI 1 not shown). Although at an MOI of 5 virus particles were detected across all harvest points in the C6/36.*w*MelPop-CLA line, at the low MOI of 0.1 no infectious particles were detected at all for days 4 and 6 post-infection (Figure 3B). Results from Wilcoxon rank sum tests indicated *Wolbachia* had a highly significant effect on viral titer for all four days post-infection that were sampled (all *p*-values <0.001). The highest amount of infectious virus was found at day 2 post-infection, suggesting detection of a few virus particles remaining from the initial inoculum or that a number of particles had escaped the inhibitory effect of *Wolbachia*. The first explanation is most likely since under the second hypothesis a steady increase in virus from days 4 to 8 would be expected rather than the decline observed (Figure 3B).

## Discussion

We have shown that infection of insect cells in culture with *Wolbachia* resulted in reduced host cell proliferation but afforded greater survival to cells when they were challenged with DENV-2. *Wolbachia* infection here had a negative effect on host cell replication, probably due to this obligate bacterium's need for host resources [28] and the high densities to which the *w*MelPop strain replicates in flies and mosquitoes [8], [18], [21], [23]. Reduced cell replication was particularly pronounced in the C6/36.*w*MelPop-CLA line, which harbored on average three times more bacteria per cell than the RML12.*w*MelPop-CLA line. However our results also showed, for both cell lines, that when dengue virus was introduced into the system, it became beneficial for cells to host *Wolbachia* due to increased survival. The results parallel the findings of Bian et al. 2010 [9] at the whole organism level where *Wolbachia*-infected female mosquitoes survived DENV-2 infection longer than uninfected females. Our results add to the growing body of evidence that, although *Wolbachia* infection imposes a metabolic cost that can lead to lowered host fitness, in certain circumstances, such as viral infections [11], [12], [14] and nutritional stress [29], these bacteria also provide a fitness benefit.

Infection with *Wolbachia* resulted in significantly lowered viral replication in the two mosquito cell lines compared to uninfected controls. Our results parallel those found in whole organisms, whereby *Wolbachia*-infected flies [11], [12], [13] and mosquitoes [8], [9] showed decreased replication of a range of RNA viruses. The same phenotype (a reduction in viral output) is observed at both the organismal and cellular levels, suggesting that the cell line models we have developed here will be useful in dissecting the functional basis of *Wolbachia*-mediated antiviral protection. Although cell line models infected with other strains of *Wolbachia* exist (e.g. *w*AlbB in *Ae. albopictus* Aa23 cells [30]; *w*Mel in Aa23 and C6/36 cells, [22]), it is unclear whether the same pattern of viral inhibition has been observed. DENV-2 replication was reduced in both cell lines infected with *w*MelPop-CLA, however, the extent of the reduction was highest in the line most densely infected with *Wolbachia*, C6/36.*w*MelPop-CLA. Viral titers in this line were dramatically reduced by almost four logs, to almost complete absence for the lowest MOI of 0.1. The difference in viral output suggests that the density of bacteria per cell may have had a significant effect on the extent of viral inhibition.

Previous work utilizing *Drosophila simulans* has suggested that *Wolbachia* strains that grow to high density provide the highest protection from virus infection [13]. It remained unclear, however, if this relationship was due to the density of *Wolbachia* or the phylogenetic history of those strains, which were most closely related to *w*Mel [13]. Our simpler, more homogenous cell line models indicate that differing densities of the same *Wolbachia* strain can modulate the extent of viral infection, with more densely infected cells displaying the strongest DENV-2 inhibition. These results favor the hypothesis that viral inhibition may be due to competition between *Wolbachia* and DENV-2 for host cellular resources [8], [14].

The reason for *Wolbachia* density differences between the two cells lines is unclear, although a similar pattern has been observed in RML12 and C6/36 cells infected with the *w*Mel *Wolbachia* strain [22]. It may be due to differences in host genetic background between the cell lines and/or the longer time since the initial establishment of *Wolbachia* infection (263 passages) for the RML12.*w*MelPop-CLA compared to the C6/36.*w*MelPop-CLA line (18 passages). Previous research has shown that although the *w*MelPop strain replicates to unusually high densities in host tissues [8], [16] its virulence attenuates under strong selection [18], [23]. Further work will be required to determine whether the C6/36.*w*MelPop-CLA line will retain a high density of *Wolbachia* during future passages and continue to display the same extent of viral inhibition.

Previous work has indicated exclusion of DENV-2 from both mosquito tissues and cells that were infected with *w*MelPop-CLA [8]. Our results suggested that exclusion of DENV-2 most likely occurred only in the very densely *Wolbachia*-infected line C6/36.*w*MelPop-CLA. Proliferation data showed that infection with DENV-2 had absolutely no effect on the replication of C6/36.*w*MelPop-CLA cells, suggesting a complete absence of viral replication. However, presence of viral titer in the RML12.*w*MelPop-CLA suggests that DENV-2 was also present in the cells that were infected with *Wolbachia*. It is unlikely that DENV-2 was replicating in a subset of cells uninfected with *Wolbachia* in the line RML12.*w*MelPop-CLA since FISH analyses established that there was almost 100% *Wolbachia* infection in both cell lines. In addition, *Wolbachia* of a different strain was found to co-localize with Japanese encephalitis virus particles in the salivary glands of the *Ae. albopictus* mosquito [31].

Previous work has shown that *Wolbachia*-infected mosquitoes display priming of immune system genes, possibly underpinning viral inhibition [8], [9], [10]. Arboviral infection in mosquitoes and some mosquito cell lines induces antimicrobial immune pathways such as Toll, JAK/STAT and Imd/JNK [32], [33] as well as RNAi-based defenses [34]. Some immune pathways, such as Toll and Imd/JNK, are also activated in *Drosophila* cell lines infected with *Wolbachia* [35]. Pre-activation of Imd in particular has been shown to inhibit Semliki Forest virus replication in a mosquito cell line [32]. However, recent work has suggested that pre-activation of immune genes by *E. coli* challenge has no impact on DENV-2 replication in a mosquito cell line [36]. The cell line models established here can be used in the future to determine whether *Wolbachia* stimulates the same immune pathways that modulate DENV-2 infection [36]. Our finding that viral inhibition in cell lines parallels that observed in whole insects allows us to study the role of immunity in this simpler model without the complexity of tissue and organ-specific expression of immune response found in whole insects [33], [34].

In summary, we have shown that cells infected with *Wolbachia* display inhibition of dengue virus replication, that the extent of inhibition is most likely related to bacterial density and that *Wolbachia* infection will provide a fitness benefit in some circumstances despite significant metabolic costs to the mosquito cell. Our results parallel those observed at the level of the whole organism, indicating that cell lines will provide useful models to examine the functional basis of *Wolbachia*-mediated viral inhibition and facilitate the development of novel vector control methods for insect-borne pathogens.

## Materials and Methods

### Cell line maintenance and *Wolbachia* infection

We used an RML12 cell line previously infected in 2007 with *w*MelPop-CLA [23]. A paired *Wolbachia*-free line, designated RML12.Tet, was derived by treating cells with 1 µg/ml tetracycline for 3 passages, followed by an additional 3 passages without antibiotic before the start of the experiment. RML12.*w*MelPop-CLA and RML12.Tet cells were routinely grown and passaged as in [23]. Absence of *Wolbachia* in the RML12.Tet line was confirmed by PCR and by fluorescent in situ hybridization (FISH). For PCR, cells were harvested at each of 4 passages post-tetracycline treatment in 2 x STE [0.2 M NaCl, 20 mM Tris-HCl and 2 mM EDTA (pH 8.0)] containing 0.8 mg/ml Proteinase K, incubated at 56°C for 30 min and 100°C for 15 min and followed by centrifugation at 13000 rpm for 2 min. Two µl of the supernatant were used for PCR as in [23]. For FISH, cells were grown to ∼80% confluence on chambered NUNC slides (Invitrogen), washed twice with PBS buffer and fixed for 15 min in 4% formaldehyde in PBS. Slides were then washed 3 times for 5 min with 0.1 M phosphate buffer (PB) and incubated with 100% EtOH for 5 min. Hybridization was performed using the *w*MelPop-specific 16s rRNA probes W2 and W3 [8]. Visualization was performed with a Zeiss Axioscope epifluorescence microscope.

C6/36 cells were infected with *w*MelPop-CLA obtained from the RML12.*w*MelPop-CLA line (above). *Wolbachia* were purified from confluent cells grown in four 175-cm^2^ by two rounds of centrifugation at 1000 g for 10 min at 4°C and resuspension in SPG buffer [23]. The suspension was filtered twice through 5 µM syringe filters and centrifuged at 13800 g for 15 min at 4°C. *Wolbachia*-enriched pellets were resuspended in 1 mL of SPG buffer and 300 µl carefully overlaid on each of three wells of C6/36 cells grown in 12-well plates to ∼80% confluence. Plates were centrifuged for 1 h at 26°C, followed by overnight incubation and cells passaged into fresh media the following day. *Wolbachia* infection levels were checked after several passages using PCR and FISH as above. Stable infection with *w*MelPop-CLA was achieved within 6 passages and remained at ∼100% throughout the course of the experiments, as checked by FISH and cell counts. Cells were routinely grown in RPMI 1640 (Invitrogen) supplemented with 1 x Glutamax (Invitrogen) and 10% FBS and buffered with 25 mM HEPES (Sigma-Aldrich). Stable infection with *w*MelPop-CLA was achieved within 6 passages and remained at ∼100% throughout the course of the experiments, as checked by FISH and cell counts. *Wolbachia* infection rates for both C6/36.wMelPop-CLA and RML12.*w*MelPop-CLA cell lines were determined as the proportion of cells displaying a fluorescent signal for the W2 and W3 probes in FISH experiments. FISH experiments were performed using 8-chambered slides, with 2 replicates of 100 cells each inspected per chamber. Infection rates are given as average percentages (± standard error).

### Determination of *Wolbachia* density by qPCR

Quantitative PCR (qPCR) was used to test for density differences in *Wolbachia* between the C6/36.*w*MelPop-CLA and RML12.*w*MelPop-CLA lines. DNA was extracted from five biological replicate samples of 250,000 cells per cell line, using the Qiagen DNAEasy kit (Qiagen). Two qPCR methods were used: 1) relative quantification of the *wsp* gene versus a reference mosquito gene *RpS17* (Cook et al. 2007); and 2) absolute quantification of the number of *wsp* gene copies per cell relative to a known standard [8]. For the first method, separate qPCR reactions were conducted for each gene, with each reaction consisting of 5 µl of SYBR Greener master mix (Invitrogen), 5 ng of DNA and 1 µM of primer in a final 10 µl volume as in [8]. Three technical replicates were performed for each of the five biological replicates per each cell line. *Wolbachia*-gene to reference-gene ratios were obtained following the method in [37]. For the second method, a standard curve was created using a cloned *wsp* gene fragment [8] with serially diluted known concentrations assayed in parallel with the samples during the qPCR.

### Cell proliferation assays

Cells were seeded in 96-well plates at 2×10^4^ cells/well and allowed to attach for 3 hours. DENV-2 infected cells were inoculated with virus at an MOI of 5, after which viral inoculum was removed 2 h post-infection and cells grown in media containing 2% FBS. Cell proliferation was assayed at 2, 4, 6 and 8 days post-infection virus using CellTiter 96® AQ_ueous_ One Solution (Promega) according to the manufacturer's instructions. Absorbance at 490 nm (directly proportional to amount of cell proliferation) was read on a SpectraMax 250 plate reader. Between 5–8 replicate wells were used for each cell line and day of assay combination as well as for DENV uninfected controls.

### Virus propagation and titration

The New Guinea C strain of DENV-2 was propagated for experimental purposes in C6/36 cells grown in media as above but supplemented with 2% FBS. Virus was harvested by collection of supernatants 7 days post-infection and centrifugation at 3200 g for 15 min at 4°C. Virus stocks were stored at −80°C in single-use aliquots and titrated on Vero cells using plaque assays. Briefly, Vero cells were seeded in 96-well plates and grown at 37°C in a humidified atmosphere with 5% CO_2_ in DMEM (Invitrogen) supplemented with 5% FBS and 1 x Glutamax. At confluence, cells were inoculated with virus and grown in Medium 199 (Invitrogen) containing 1% carboxymethylcellulose and supplemented with 2% FBS. Five days post-infection, cell monolayers were washed with PBS, fixed with acetone/PBS, blocked for 1 h and incubated for another hour with a primary antibody against the DENV-2 NS1 protein (1∶500). Monolayers were washed with PBS, incubated for 1 h with a conjugated horseradish peroxidase (1∶500), followed by a final brief incubation with SIGMA*Fast* DAB (D0426, Sigma-Aldrich) until plaques became visible.

### Experimental determination of DENV-2 replication

Viral replication experiments were performed using both pairs of *Wolbachia* infected and uninfected cell lines, with each pair of lines as a separate experiment. Cells were plated at 4×10^5^ cells/well in 12-well plates and allowed to attach for 3 hours in media with 10% FBS. Cells were then infected with virus in FBS-free media at MOI of 0.1, 1 and 5. The viral inoculum was removed 2 h post-infection and cells were maintained in 2 mL of media with 2% FBS. Viral harvests occurred at 2, 4, 6 and 8 days post-infection, with supernatants from each well clarified from cells by centrifuging for 10 min at 4000 g at 4°C and frozen at −80°C until titration. Experiments were performed in triplicate wells for each MOI, cell line (*Wolbachia*-infected or uninfected) and harvest day combination. Titrations were performed in duplicate for each supernatant using plaque assays.

### Statistical analysis

Cell proliferation data for each pair of lines was analyzed as a three-way ANOVA with presence/absence of *Wolbachia*, virus infected/uninfected, day post-infection and their interactions. The effects of *Wolbachia* and virus infection were also explored for each day post-infection using two-way ANOVAs. To assess differences in *Wolbachia* density between the C6/36.*w*MelPop and RML12.*w*MelPop lines, gene expression ratio data obtained from qPCRs was analyzed with a two-sample Wilcoxon rank sum test. We used two-sample t-tests to detect statistically significant differences in the absolute average density of *Wolbachia* between the two cell lines.

Plaque assay data from the RML12.Tet and RML12.*w*MelPop cell lines was log-transformed to fit the assumptions of parametric analyses. Three-way ANOVAs were initially performed with the main factors presence/absence of *Wolbachia*, MOI, day post-infection and their interactions. Since interactions were found to be statistically significant (results not shown) thus rendering the interpretation of main effects problematic, data was pooled across MOIs within each day. Two-sample t-tests were performed on the pooled data for each day to test for the effect of *Wolbachia* on viral titer. Although plaque assay data obtained from the C6/36 and C6/36.*w*MelPop lines was log-transformed it still strongly deviated from normality. Results from a generalized linear model for non-normal data indicated that the presence/absence of *Wolbachia*, the day post-infection, MOI and the interaction between the three factors had a significant effect on viral titer (results not shown). Consequently, data was pooled across MOIs within days and nonparametric Wilcoxon rank sum tests were performed for each day to test for the effect of *Wolbachia* on viral titer. All analyses were implemented in R [38].
